# Financial Incentives to Facilities and Clinicians Treating Patients With End-stage Kidney Disease and Use of Home Dialysis: A Randomized Clinical Trial

**DOI:** 10.1001/jamahealthforum.2022.3503

**Published:** 2022-10-07

**Authors:** Yunan Ji, Liran Einav, Neale Mahoney, Amy Finkelstein

**Affiliations:** 1 McDonough School of Business, Georgetown University, Washington, DC; 2 Department of Economics, Stanford University, Stanford, California; 3 National Bureau of Economic Research, Cambridge, Massachusetts; 4 J-PAL North America, Cambridge, Massachusetts; 5 Department of Economics, Massachusetts Institute of Technology, Cambridge

## Abstract

**Importance:**

Home dialysis rates for end-stage kidney disease (ESKD) treatment are substantially lower in the US than in other high-income countries, yet there is limited knowledge on how to increase these rates.

**Objective:**

To report results from the first year of a nationwide randomized clinical trial that provides financial incentives to ESKD facilities and managing clinicians to increase home dialysis rates.

**Design, Setting, and Participants:**

Results were analyzed from the first year of the End-Stage Renal Disease Treatment Choice (ETC) model, a multiyear, mandatory-participation randomized clinical trial designed and implemented by the US Center for Medicare & Medicaid Innovation. Data were reported on Medicare patients with ESKD 66 years or older who initiated treatment with dialysis in 2021, with data collection through December 31, 2021; the study included all eligible ESKD facilities and managing clinicians. Eligible hospital referral regions (HRRs) were randomly assigned to the ETC (91 HRRs) or a control group (211 HRRs).

**Interventions:**

The ESKD facilities and managing clinicians received financial incentives for home dialysis use.

**Main Outcomes and Measures:**

The primary outcome was the percentage of patients with ESKD who received any home dialysis during the first 90 days of treatment. Secondary outcomes included other measures of home dialysis and patient volume and characteristics.

**Results:**

Among the 302 HRRs eligible for randomization, 18 621 eligible patients initiated dialysis treatment during the study period (mean [SD] age, 74.8 [1.05] years; 7856 women [42.1%]; 10 765 men [57.9%]; 859 Asian [5.2%], 3280 [17.7%] Black, 730 [4.3%] Hispanic, 239 North American Native, and 12 394 managing clinicians. The mean (SD) share of patients with any home dialysis during the first 90 days was 20.6% (7.8%) in the control group and was 0.12 percentage points higher (95% CI, −1.42 to 1.65 percentage points; *P* = .88) in the ETC group, a statistically nonsignificant difference. None of the secondary outcomes differed significantly between groups.

**Conclusions and Relevance:**

The trial results found that in the first year of the US Center for Medicare & Medicaid Innovation–designed ETC model, HRRs assigned to the model did not have statistically significantly different rates in home dialysis compared with control HRRs. This raises questions about the efficacy of the financial incentives provided, although further evaluation is needed, as the size of these incentives will increase in subsequent years.

**Trial Registration:**

ClinicalTrials.gov Identifier: NCT05005572

## Key Points

**Question:**

How did the End Stage Renal Disease Treatment Choice model affect home dialysis rates?

**Findings:**

In this randomized clinical trial of 18 621 patients with end-stage kidney disease, in an analysis of the first year of the End Stage Renal Disease Treatment Choice model that randomly assigned 91 hospital referral regions to treatment and 211 to control, the mean percentage of patients with any home dialysis during the first 90 days was not statistically significantly different in the treatment and control groups.

**Meaning:**

The trial found no statistically significantly different rates of home dialysis between treatment and control hospital referral regions during the first year of the program, suggesting that higher incentives may be necessary to change clinician and facility behavior.

## Teaser

This randomized clinical trial examines financial incentives to end-stage kidney disease facilities and managing clinicians to increase home dialysis rates.

## Introduction

Patients with end-stage kidney disease (ESKD) comprise 1% of traditional Medicare enrollment but 7% of its spending.^1,2^ The most common treatment for ESKD is dialysis, which filters a patient’s blood of waste and toxins. Dialysis may be done at home, typically requiring daily overnight sessions while asleep, or in a facility, typically requiring 2 to 3 patient visits per week. Home dialysis is associated with lower spending and equal or better clinical outcomes than facility-based dialysis.^3,4,5,6^ It has been estimated that up to 85% of patients may be suitable to receive home dialysis.^7^

Yet, in 2019, only 12.6% of Medicare patients receiving dialysis underwent dialysis at home.^8^ By contrast, in several countries, including Australia, Canada, Denmark, Hong Kong, and New Zealand, rates of home dialysis range from one-quarter of patients to more than half.^8^ In 2019, the US Department of Health and Human Services announced a goal of having 80% of new patients with ESKD either receive dialysis at home or receive a transplant by 2025.^2^

The choice between home and facility dialysis reflects a combination of clinical, social, and/or financial considerations. From a clinical perspective, patients with multiple comorbidities are usually considered more appropriate for facility-based dialysis.^9^ Some patients may also appreciate the assistance that ESKD facilities provide during treatment sessions, while others may prefer receiving dialysis at home, which allows them to cut back on travel to dialysis facilities.^10,11,12,13^

Finally, financial incentives may encourage managing clinicians and ESKD facilities to steer patients away from home dialysis. The monthly Medicare physician fees from treating a home dialysis patient are on average more than 10% lower than those for patients receiving dialysis in a facility.^14^ Facilities can use the same machine across multiple patients who receive facility-based dialysis, but cannot when patients receive dialysis at home.^15^ These financial considerations may not necessarily align with benefits to the patient. Prior observational studies have suggested that increased payments for home dialysis relative to facility dialysis may increase home dialysis rates.^16,17,18^

As part of the attempt to increase rates of home dialysis and kidney transplant to meet the 80% goal, the US Center for Medicare & Medicaid Innovation (CMMI) designed and implemented a new payment model that increased financial incentives for ESKD facilities and managing clinicians.^2^ The model, End-Stage Renal Disease Treatment Choice (ETC), is one of more than 50 payment models developed and implemented by CMMI since its founding in 2010.^19^ It is one of only 5 of these models to date that were implemented as mandatory participation randomized clinical trials. The ETC began on January 1, 2021, and the last measurement period ends on June 30, 2026.^20^ The purpose of this study was to analyze results from the first year of the ETC program, which was calendar year 2021.

## Methods

Institutional review board (IRB) exemption was obtained from the Massachusetts Institute of Technology’s Committee on the Use of Humans as Experimental Subjects, Harvard University IRB, and Stanford University IRB. All IRBs waived informed consent for the analysis because of minimal risk. The CMMI did not require informed consent for patients in ETC. All reported analyses were prespecified (before obtaining postintervention data) unless explicitly designated otherwise (Supplement 1). This study follows the Consolidated Standards of Reporting Trials (CONSORT) reporting guideline for randomized clinical trials.

### Study Design

On July 10, 2019, CMMI announced the ETC model, a cluster trial with randomization at the hospital referral region (HRR) level, with a proposed start date of January 1, 2020; this was later delayed to January 1, 2021.^21^ On September 18, 2020, CMMI assigned 30% of HRRs to treatment through stratified randomization by census regions (Northeast, South, Midwest, and West). However, HRRs with 20% or more of their zip codes in Maryland were directly assigned to treatment, and US territories were excluded from treatment; we excluded all of these HRRs from the analysis (Figure).

**Figure.**
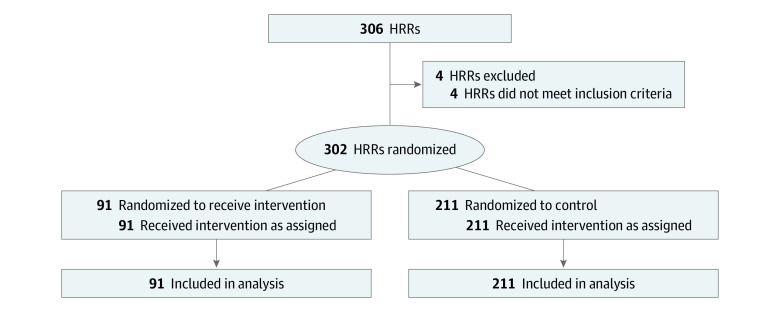
CONSORT Diagram of Hospital Referral Region (HRR) Eligibility and Randomization in End-Stage Renal Disease Treatment Choice (ETC)

Within eligible HRRs randomized to ETC, all ESKD facilities and clinicians were required to participate; subsequently, facilities and clinician groups with fewer than 11 attributed Medicare patients with ESKD in a performance year were excluded. Patients with ESKD were excluded from treatment if at any point during the measurement period the patient was only receiving dialysis for acute kidney injury; was younger than 18 years; received a kidney transplant within the past 12 months and did not experience transplant failure; was not enrolled in Medicare Part B; was enrolled in Medicare Advantage, a cost plan, or other Medicare managed care plan; did not reside in the US; elected hospice; received a diagnosis of dementia; or was residing or receiving dialysis in a skilled nursing facility.

Our analysis closely followed the eligibility criteria for the ETC model. We excluded all the facilities, clinicians, and patients that would not be eligible for the model with 1 exception: we did not exclude facilities or clinician groups with fewer than 11 attributed patients because we are unable to accurately identify clinician groups in the data. We made 2 additional sample restrictions. First, we focused on patients who were new to dialysis, defined as not having received dialysis for at least 12 months before the first observed dialysis claim. We applied this restriction to help improve statistical power, because we suspected the program would primarily affect new patients rather than changing the treatment modality of existing patients, although in eTable 6 in Supplement 2 we show robustness of the results to removing this restriction. Second, we restricted to patients who were at least age 66 years during the first month of dialysis to ensure that we observed at least 12 months of Medicare claims before their first dialysis. The eAppendix in Supplement 2 provides more detail on the program eligibility and our study sample. eTable 1 in Supplement 2 reports the resulting sample sizes after applying each sample restriction.

### Intervention

The ETC model makes 2 types of payment adjustments. The first adjustment, home dialysis payment adjustment (HDPA), increases the reimbursement rate for home dialysis for the first 3 years of the ETC program. The second adjustment, performance payment adjustment (PPA), increases or decreases the reimbursement rate for home and facility dialysis based on the rate of home dialysis and transplant, which are combined into a modality performance score. Both payment adjustments apply to ESKD facilities and managing clinicians. Peritoneal and home hemodialysis are included when computing home dialysis rates.

The HDPA increase in reimbursement rate for home dialysis was 3% in 2021, 2% in 2022, and 1% in 2023. The PPA adjustments were potentially much larger and depended on the facility’s modality performance score. They could range from −5% to 4% in the first year and increase over time to −10% to 8% during the last year of the program. The first year of PPA was calendar year 2021, and performance in that year affected reimbursement rates between July and December 2022.

The magnitude of the PPA adjustment depended on a modality performance score. This was computed as a weighted average of the facility’s home dialysis rate (including home dialysis and in-facility self-dialysis) and transplant rate (measured by patients who received live donor transplants and patients on the wait list for them), with home dialysis rate receiving twice the weight as the transplant rate. For the home dialysis and transplant rates, scores were computed as the higher of an achievement score (percentile of score relative to ESKD facilities and managing clinicians in the control HRRs during the benchmark year, which was July 1, 2019, to June 30, 2020, for the first year) and an improvement score (percent improvement relative to own performance during the benchmark year). The eAppendix in Supplement 2 provides more detail on the construction of the modality score.

### Data and Outcome Measures

We studied the first year of the ETC program, which began on January 1, 2021. The study sample included all new patients who initiated treatment with dialysis for ESKD between January 1, 2021, and October 3, 2021; the end date was chosen to ensure that we could observe all subsequent claims and outcomes of these patients for 90 days.

We used Medicare fee-for-service claims data covering 100% of Medicare beneficiaries from 2018 and 2021 in the 302 HRRs eligible for randomization. We restricted the sample to facilities, managing clinicians, and beneficiaries who would have been covered by ETC if the HRR were selected for treatment. We omitted data from 2019 and 2020 because the ETC program was first announced in 2019, and clinicians and facilities could have started changing behavior in anticipation of the program. This was particularly likely because the first benchmark year used for computing PPA adjustments was July 1, 2019, to June 30, 2020. In addition to the baseline sample, we also created subsamples by patient and facility characteristics to explore heterogeneity in the effect. Patient race and ethnicity was identified based on the Medicare Enrollment Database and included the following categories: Asian, Black, Hispanic, North American Native, White, and Other (including beneficiaries who did not identify as any of the previously mentioned racial and ethnic groups and beneficiaries who did not respond).

The primary outcome was the percentage of patients receiving any home dialysis during the first 90 days since the start of dialysis treatment. The incentivized transplant rate, which included patients on the wait list, was not measurable in the Medicare claims data. The realized transplant component was measurable, but rare (about 1% in 2019); our power calculations suggested we would be unable to detect meaningful changes in this measure.

Secondary outcomes included alternative measures of home dialysis utilization: the percentage of weeks a patient received any home dialysis in the first 90 days and percentage of dialysis sessions at home in the first 90 days. Other secondary outcomes included measures of patient characteristics (predialysis Elixhauser index score) and extensive margin outcomes (new dialysis patients per capita and total number of dialysis patients). We also investigated possible anticipatory effects by analyzing the primary outcome in calendar year 2020, which was after the announcement but before the implementation of the ETC model. We did not analyze downstream outcomes, such as hospitalizations or mortality, because we lacked power to detect meaningful changes. The eAppendix in Supplement 2 provides more detail on data and outcomes.

### Statistical Analyses

We conducted the statistical analyses at the HRR level, as it was the level of randomization. We estimated ordinary least squares regressions of the outcome of interest on an indicator for whether the HRR was assigned to treatment and covariates. We included census region fixed effects as covariates since randomization was stratified by census regions. To improve statistical power, we also controlled for the lag of the HRR-level outcome from 2018, as well as HRR-level demographic characteristics (share of patients in each 5-year age bin, share of White patients, and share of female patients) and the HRR average of each of the 31 Elixhauser chronic conditions, which were measured 12 months before dialysis. We measure the lagged outcome in 2018 to avoid anticipatory effects from the ETC program announcement in 2019. We weighted each HRR by the average number of patients in the sample from 2018 to 2019.

The power calculation based on historical data suggested that we had power to detect a 1.98 percentage point change in the primary outcome (2-sided α = .05 at 80% power), which was on par with the US Centers for Medicare and Medicaid Services Office of the Actuary’s forecasted annual increase in home dialysis rates.^20^ An effect of this magnitude seemed reasonable given the large potential for increased home dialysis use (relative to the share of eligible patients and rates in other countries), as well as the implementation of the ETC model in service of the administration’s goal of achieving 80% home dialysis or transplant by 2025.

We performed a series of sensitivity and heterogeneity analyses, including analyses of 30-day rather than 90-day outcomes, using alternative covariates and different definitions of home dialysis. We also performed 2 analyses that were not prespecified: we included all patients receiving dialysis for ESKD treatment in the analysis sample, rather than just new patients, and we analyzed heterogeneity in effects by home dialysis rates in the benchmark year (which affects the magnitude of the program incentives).

All statistical analyses were conducted using Stata, version 16 (StataCorp). The eAppendix in Supplement 2 provides more detail.

## Results

### Study Population

The eFigure in Supplement 2 shows the geographic distribution of the treatment and control HRRs. Within these 302 HRRs, 6000 facilities and 18 621 patients met the sample eligibility criteria. Table 1 describes the study population in the control HRRs. Patients had a mean (SD) age of 74.8 (1.05) years; there were 7856 women (42.1%) and 10 765 men (57.9%); and there were 859 Asian (5.2%), 3280 (17.7%) Black, 730 (4.3%) Hispanic, 239 North American Native, and 12 394 (65.7%) White individuals. A total of 3323 (18%) had disabilities, 4502 (25%) were Medicaid beneficiaries, and 15 328 (83%) resided in an urban area. The average patient had 6 Elixhauser chronic conditions and lived 5.2 miles from the nearest dialysis facility. A total of 1530 dialysis facilities (89%) were for profit and 1125 (66%) were part of a chain. The average HRR had 42 dialysis facilities per 1000 Medicare fee-for-service beneficiaries who receive dialysis.

**Table 1.** Characteristics of the Control HRRs in 2021^a^

Characteristic	HRR level	
	Mean (SD)	Median (IQR)
Patients receiving dialysis		
Mean (SD) age, y	74.80 (1.05)	74.78 (74.10-75.60)
Asian, %	5.18 (7.54)	2.17 (0-5.88)
Black, %	17.74 (14.21)	13.04 (6.78-25.97)
Hispanic, %	4.30 (6.09)	1.70 (0-5.71)
North American Native, %	0.86 (3.98)	0
White, %	65.67 (18.21)	69.33 (50.57-78.11)
Other, %^b^	6.26 (6.51)	5.06 (2.38-8.13)
Female, %	42.08 (6.63)	42.15 (37.93-46.07)
Male, %	57.92 (6.63)	57.85 (53.93-62.07)
With disabilities, %	17.53 (6.28)	17.06 (13.52-20.00)
Medicaid beneficiaries, %	25.37 (15.56)	20.06 (14.70-28.35)
Urban, %	83.11 (19.51)	88.37 (75.00-100.00)
Average Elixhauser Comorbidity Index score	5.96 (0.75)	6.02 (5.35-6.41)
Average distance-nearest dialysis facility	5.23 (3.48)	4.51 (2.99-6.74)
Dialysis facilities		
ETC-eligible dialysis patients per facility^c^		
All ages	35.69 (9.07)	34.83 (29.33-41.71)
≥66 y^d^	16.53 (5.13)	15.64 (13.08-19.31)
≥66 y^e^	2.77 (0.93)	2.71 (2.08-3.31)
For profit, %	88.91 (15.76)	96.00 (84.62-100.00)
Chains (Fresinius or DaVita), %	65.63 (19.09)	68.09 (55.56-78.13)
HRRs		
Dialysis facilities per 1000 beneficiaries^f^	41.73 (10.00)	41.07 (33.67-49.10)

Abbreviations: ETC, End-Stage Renal Disease Treatment Choice; HRRs, hospital referral regions.

^a^ Table reports characteristics of patients, facilities, and 221 HRRs in the control group that met the baseline sample definition in 2021. All summary statistics are reported at the HRR level and are weighted by the average number of patients in the sample in 2018 and 2019.

^b^ Includes patients who did not identify as any of the racial and ethnic groups included in the table and patients whose race was unknown.

^c^ Without the baseline simple restriction that patients must be new to treatment with dialysis and 66 years or older.

^d^ Without the baseline sample restriction that patients must be new to treatment with dialysis.

^e^ Includes only patients in the baseline sample: traditional Medicare beneficiaries 66 years or older who initiated treatment with dialysis between January 1 and October 3, 2021.

^f^ Number of dialysis facilities in the HRR with at least 1 claim between January 1 and October 3, 2021, divided by the number of traditional Medicare beneficiaries 66 years or older who resided in the HRR in 2021.

### Balance

Characteristics of treatment and control HRRs were balanced in 2018 before randomization (Table 2). An *F* test failed to reject equality of all the outcomes (*F* statstic, 1.08; *P *= .11).

**Table 2.** Balance of Study Population by Group in 2018 Before Announcement of ETC^a^

Characteristic	Value in control HRRs, mean (SD)	Between treatment and control HRRs, mean difference (95% CI)	*P* value
Treatment modality			
Any home dialysis in first 90 d, %	16.52 (8.49)	−0.389 (−2.12 to 1.35)	.66
Weeks receiving any home dialysis in first 90 d, %	13.67 (7.37)	−0.48 (−2.19 to 1.22)	.58
Dialysis sessions at home in first 90 d, %	13.96 (7.34)	−0.51 (−2.17 to 1.16)	.55
Patient characteristics and extensive margin outcomes			
Dialysis rate per capita	0.01 (0.01)	0.0002 (0)	.10
Total No. of dialysis patients	2946 (3055)	87.49 (5.60 to 169.39)	.04
Predialysis Elixhauser index score	5.47 (0.83)	0.09 (0 to 0.19)	.05
Heterogeneity by patient characteristics			
Any home dialysis in first 90 d, %			
Non-Medicaid^b^	19.55 (9.14)	−0.123 (−2.14 to 1.90)	.91
Medicaid^b^	7.78 (9.12)	0.58 (−1.56 to 2.73)	.60
White	18.70 (9.88)	0.382 (−1.92 to 2.68)	.75
Other racial and ethnic minority groups^g^	12.03 (10.47)	−0.067 (−2.80 to 2.66)	.97
Rural^c^	13.20 (13.64)	−1.14 (−4.58 to 2.30)	.52
Urban^c^	17.97 (10.77)	−0.55 (−2.69 to 1.60)	.62
Without disabilities^d^	17.47 (9.33)	−0.42 (−2.34 to 1.51)	.68
With disabilities^d^	12.81 (12.72)	−0.056 (−2.91 to 2.80)	.97
Median distance to nearest facility^e^			
Greater than	18.11 (9.90)	1.27 (−1.17 to 3.72)	.31
Less than	15.44 (11.11)	−2.42 (−4.83 to −0.01)	.05
Heterogeneity by facility characteristics			
Any home dialysis in first 90 d, %^f^			
Nonprofit	12.34 (15.51)	−2.84 (−8.51 to 2.84)	.33
For profit	16.33 (9.50)	−0.399 (−2.35 to 1.55)	.69
Nonchains	15.84 (13.07)	−0.15 (−3.45 to 3.15)	.93
Chains (Fresinius or DaVita)	16.00 (10.02)	−0.4515 (−2.92 to 2.02)	.72
Median volume^h^			
Above	16.68 (10.88)	−0.0651 (−2.26 to 2.13)	.96
Below	16.64 (12.20)	0.871 (−3.21 to 4.95)	.68

Abbreviations: ETC, End-Stage Renal Disease Treatment Choice; HRRs, hospital referral regions; RUCA, rural-urban commuting area codes.

^a^ The table reports HRR-level average characteristics of patients in the baseline sample in 2018, the year before the announcement of ETC. The first column reports the means for the control HRRs. The second column reports the coefficient on the treatment dummy from estimating an HRR-level regression of the outcome variable on the indicator for treatment, controlling for strata fixed effects, lagged outcomes from t3 years prior, and HRR-level averages of patient demographic characteristics and baseline health. The regression is weighted by the average number of patients in the sample in 2018 and 2019. We report 95% CIs based on heteroskedasticity robust standard errors. Medians are computed at the national level. An *F *test of hypothesis that all of the outcomes are jointly zero yielded an *F *statistic of 1.08 and *P* = .11.

^b^ Receiving Medicaid anytime during the first 90 days of treatment.

^c^ Based on the RUCAs. Zip codes with an RUCA of 4 or greater are defined as rural. All other zip codes are defined as urban.

^d^ Whether the original reason for Medicare entitlement includes disability.

^e^ The geodetic distance from the centroid of the 5-digit zip code for the patient to the exact address of the facility.

^f^ Based on the 2021 Provider of Services file.

^g^ Includes patients who did not identify as any of the previously described groups and patients whose race was unknown.

^h^ Includes all Medicare dialysis patients in 2018, not just the baseline sample.

### ESKD Treatment Modality, Patient Characteristics, and Extensive Margin Outcomes

Table 3 reports the effect of the ETC based on the prespecified fully adjusted regression model. The primary outcome (percentage of new patients with any home dialysis in the first 90 days) had a mean (SD) of 20.60% (7.77%) in the control group and was 0.12 percentage points higher (95% CI, −1.42 to 1.65; *P* = .89) in the treatment group, a statistically nonsignificant difference.

**Table 3.** Effect of ETC During First Year of the Program in 2021^a^

Characteristic	Value in control HRRs, mean (SD)	Between treatment and control HRRs, mean difference (95% CI)	*P* value
Treatment modality			
Any home dialysis in first 90 d, %	20.60 (7.77)	0.12 (−1.42 to 1.65)	.89
Weeks receiving any home dialysis in first 90 d, %	16.67 (6.77)	0.17 (−1.24 to 1.58)	.82
Dialysis sessions at home in first 90 d, %	17.23 (6.81)	0.22 (−1.14 to 1.57)	.76
Patient characteristics and extensive margin outcomes			
Dialysis rate per capita^b^	0.01 (0.005)	−0.0001 (−0.0003 to 0.0002)	.44
Total No. of dialysis patients^c^	2388 (2521)	37.04 (−8.41 to 82.50)	.11
Predialysis Elixhauser index score	5.96 (0.75)	−0.02 (−0.18 to 0.13)	.77
Anticipatory effect			
Any home dialysis in first 90 d in 2020, %	20.00 (8.55)	−1.20 (−2.75 to 0.3382)	.13

Abbreviations: ETC, End-Stage Renal Disease Treatment Choice; HRRs, hospital referral regions.

^a^ The table reports HRR-level average characteristics of ETC-eligible patients. The first column reports the means for the control HRRs. The second column reports the coefficient on the treatment indicator from estimating an HRR-level regression of the outcome variable on the treatment indicator, controlling for strata fixed effects, lagged outcome from 3 years prior, and HRR-level averages of patient demographic characteristics and baseline health. The regression is weighted by the average number of patients in the sample in 2018 and 2019. We report 95% CIs based on heteroskedasticity robust standard errors.

^b^ This is the number of traditional Medicare patients 66 years or older who initiated treatment with dialysis in either modality in the baseline sample divided by the number of traditional Medicare patients 66 years or older.

^c^ Includes all traditional Medicare patients who received dialysis between January 1 and October 3, 2021.

We detected no statistically significant difference in the secondary outcomes. The mean (SD) percentage of weeks a patient received any home dialysis during the first 90 days was 16.67% (6.77%) in the control group and 0.17 percentage points higher (95% CI, −1.24 to 1.58; *P* = .82) in the treatment group. The mean (SD) percentage of dialysis sessions at home during the first 90 days was 17.23% (6.81%) in the control group and 0.22 percentage points higher (95% CI, −1.14 to 1.57; *P* = .75) in the treatment group. There were no statistically significant differences between the treatment and control groups in patient characteristics or volume, nor was there any evidence of anticipatory effects. We also found no effect of the intervention when we used alternative controls or without controls (eTable 2 in Supplement 2), when we used alternative outcome definitions (eTables 4-5 in Supplement 2), and when we expanded the sample to include all Medicare dialysis patients, rather than only new dialysis patients 66 years or older (eTable 6 in Supplement 2).

### Heterogeneity

We found no statistically significant difference between treatment and control groups in the percentage of patients with any home dialysis during the first 90 days when we looked at various patient subsamples (Table 4). These included analyses by patient characteristics, such as Medicaid status, race and ethnicity, urbanity, and distance to the nearest facility, as well as facility characteristics, such as ownership type or chain status. We found similar results when using alternative controls or without controls (eTable 3 in Supplement 2). We also found no effect of the intervention on the primary or secondary outcomes by the heterogenous incentives across facilities with different baseline modality performance scores (eTable 7 in Supplement 2).

**Table 4.** Heterogeneity in Effect on Home Dialysis^a^

Characteristic	Value in control HRRs, mean (SD)	Between treatment and control HRRs, mean difference (95% CI)	*P* value	*P* value of difference
**Outcome: percentage of any home dialysis in first 90 d**				
Heterogeneity by patient characteristics				
Non-Medicaid	23.92 (8.36)	−0.445 (−2.18 to 1.28)	.62	.22
Medicaid	9.45 (9.34)	1.30 (−0.98 to 3.57)	.27	
White	23.28 (8.89)	−0.1101 (−1.93 to 1.70)	.91	.87
Other racial and ethnic minority groups^b^	15.05 (11.10)	−0.39 (−3.24 to 2.45)	.79	
Rural	14.83 (12.60)	0.23 (−2.94 to 3.40)	.89	.99
Urban	22.45 (9.94)	0.1895 (−1.92 to 2.30)	.87	
Without disabilities	21.75 (8.60)	−0.2259 (−1.82 to 1.37)	.79	.08
With disabilities	14.96 (10.86)	2.52 (−0.24 to 5.28)	.08	
Median distance to nearest facility				
Greater than	21.89 (10.96)	−0.91 (−3.06 to 1.24)	.41	.66
Less than	18.61 (11.63)	−0.18 (−2.74 to 2.38)	.89	
Heterogeneity by facility characteristics				
Nonprofit	16.42 (19.52)	0.769 (−5.93 to 3.46)	.83	NA
For profit	20.69 (8.72)	−0.82 (−2.83 to 1.39)	.43	.66
Nonchains	19.54 (15.42)	0.12 (−3.22 to 4.57)	.95	.37
Chains (Fresinius or DaVita)	20.86 (9.55)	−1.73 (−3.81 to 1.62)	.11	
Median volume				
Greater than	20.67 (11.27)	0.76 (−1.43 to 2.70)	.50	.19
Less than	21.64 (15.32)	−1.73 (−4.60 to −0.59)	.24	

Abbreviations: HRRs, hospital referral regions; NA, not applicable.

^a^ The table replicates Table 3 but separately for each group of patients or facilities. The *P* value in the fourth column is generated through a *t* test of the 2 coefficients in the second column after estimating each pair of regressions as seemingly unrelated regressions. Medians are computed at the national level. See Table 2 for variable definitions.

^b^ Includes patients who did not identify as any of the previously described groups and patients whose race was unknown.

## Discussion

In this randomized clinical trial and analysis of the first year of a Medicare payment reform for treatment of patients with ESKD, HRRs randomly assigned to the model did not have statistically significantly different rates of home dialysis than HRRs in the control group. The 95% CIs rule out an increase in home dialysis rates of more than 1.65 percentage points (8%), although we are unable to rule out increases less than this level. We also did not detect statistically significant differences in any secondary outcomes or subsamples.

Financial incentives have been shown to affect treatment decisions by health care clinicians and facilities in other settings, including in the provision of physician services, prescription drugs, and hospital discharge decisions.^22,23,24,25^ There is also evidence from prior observational studies of an effect of financial incentives on the dialysis modality choice of patients with ESKD. A difference-in-differences analysis of a 2004 US Centers for Medicare & Medicaid reform of physician fees found that increased payments for frequent facility-based hemodialysis sessions (the primary form of facility-based dialysis) reduced home dialysis rates among traditional Medicare patients with ESKD by 0.7 percentage points relative to the Medicare Advantage patients with ESKD who were not affected by the reform.^16^ Pre-post analyses of the introduction of a Medicare prospective payment system (PPS) for patients with ESKD, which reduced payments for facility-based dialysis relative to home dialysis, found about a 5 percentage point increased use of peritoneal dialysis (which is predominantly administered at home),^18,26^ with a larger increase among patients new to treatment with dialysis.^17^

In contrast to these observational studies, we found no statistically significant effect of the financial incentives for home dialysis in the ETC model on dialysis modality choice. One reason may be that the ETC model was used as a mandatory participation, randomized clinical trial, making it less vulnerable to confounding factors than an observational study. Another potential reason may be that the magnitude of the incentives was larger in the observational studies. For example, the introduction of PPS meant that anemia drugs required for facility-based dialysis, which comprised up to 40% of facility profits before PPS, was no longer separately billable, thus significantly reducing the profitability of facility-based dialysis relative to home dialysis.^27,28^ Likewise, following the 2004 reform, monthly physician fees for the average facility dialysis patient increased by more than 10% relative to fees for home dialysis patients.^14^ The potential payment adjustment during the first year of ETC (3% for HDPA and −4% to 5% for PPA) was more modest. However, similar or more modest payment changes in other health care settings have been found to affect clinician behavior. For instance, a study of a change in Medicare physician payments found that a 2% increase in payments led to a 3% increase in service provision.^24^ A randomized clinical trial of bundled payments for lower extremity joint replacement that provided financial incentives to hospitals that were comparable in magnitude to those in the first year of ETC found a statistically significant 2.9 percentage point reduction in institutional post–acute care use during the first year of the program.^29^

### Limitations

This study had several limitations. First, we evaluated only the first year of the ETC program, while the financial incentives become larger in later years. Second, we analyzed only changes in the home dialysis rates and not transplant rates. Third, the study was not powered to detect changes in downstream outcomes, including changes in patient health and health care use. Finally, we focused on the Medicare patients with ESKD patients 66 years or older, whose outcomes may differ from those of younger patients.

## Conclusions

In this evaluation of the first year of a randomized clinical trial of the ETC model for Medicare beneficiaries with ESKD, HRRs randomly assigned to the model did not have statistically significantly different rates in home dialysis than control HRRs. This raises questions about the efficacy of the financial incentives in the model and suggests that higher incentives may be necessary to affect behavioral change by dialysis clinicians and facilities. Further evaluation is needed as these incentives increase in subsequent program years.

## Notes

Supplement 1. Trial protocol

Supplement 2. **eFigure.** Map of HRRs by Treatment and Control Status **eTable 1.** Sample Restrictions **eTable 2.** Robustness to Alternative Controls (2021) **eTable 3. **Robustness to Alternative Controls, Heterogeneity by Patient and Facility Characteristics (2021) **eTable 4. **Impact of ETC During First Year of the Program (2021), 30-Day Outcomes **eTable 5.** Impact of ETC on Home Dialysis and Self Dialysis (2021) **eTable 6.** Impact of ETC During First Year of the Program (2021) **eTable 7. **Impact of ETC During First Year of the Program Controlling for Benchmark Year Achievement (2021) **eAppendix. **Prespecified Analysis Plan

Supplement 3. Data sharing statement
