# Supplementary material for: Financial Incentives to Facilities and Clinicians Treating Patients With End-stage Kidney Disease and Use of Home Dialysis: A Randomized Clinical Trial
Source: JAMA Health Forum. 2022 Oct 7;3(10):e223503. doi: 10.1001/jamahealthforum.2022.3503 (PMC9547325; doi:10.1001/jamahealthforum.2022.3503)
Supplement: Supplement 2. — eFigure. Map of HRRs by Treatment and Control Status eTable 1. Sample Restrictions eTable 2. Robustness to Alternative Controls (2021) eTable 3. Robustness to Alternative Controls, Heterogeneity by Patient and Facility Characteristics (2021) eTable 4. Impact of ETC During First Year of the Program (2021), 30-Day Outcomes eTable 5. Impact of ETC on Home Dialysis and Self Dialysis (2021) eTable 6. Impact of ETC During First Year of the Program (2021) eTable 7. Impact of ETC During First Year of the Program Controlling for Benchmark Year Achievement (2021) eAppendix. Prespecified Analysis Plan [file jamahealthforum-e223503-s002.pdf]

Supplemental Online Content

Ji Y, Einav L, Mahoney N, Finkelstein A. Financial incentives to facilities and clinicians treating patients with end-stage kidney disease and use of home dialysis: a randomized clinical trial. *JAMA Health Forum*. 2022;3(10):e223503. doi:10.1001/jamahealthforum.2022.3503

eFigure. Map of HRRs by Treatment and Control Status

eTable 1. Sample Restrictions

eTable 2. Robustness to Alternative Controls (2021)

eTable 3. Robustness to Alternative Controls, Heterogeneity by Patient and Facility Characteristics (2021)

eTable 4. Impact of ETC During First Year of the Program (2021), 30-Day Outcomes

eTable 5. Impact of ETC on Home Dialysis and Self Dialysis (2021)

eTable 6. Impact of ETC During First Year of the Program (2021)

eTable 7. Impact of ETC During First Year of the Program Controlling for Benchmark Year Achievement (2021)

eAppendix. Prespecified Analysis Plan

This supplemental material has been provided by the authors to give readers additional information about their work.

© 2022 Ji Y et al. *JAMA Health Forum.*

eFigure. Map of HRRs by Treatment and Control Status

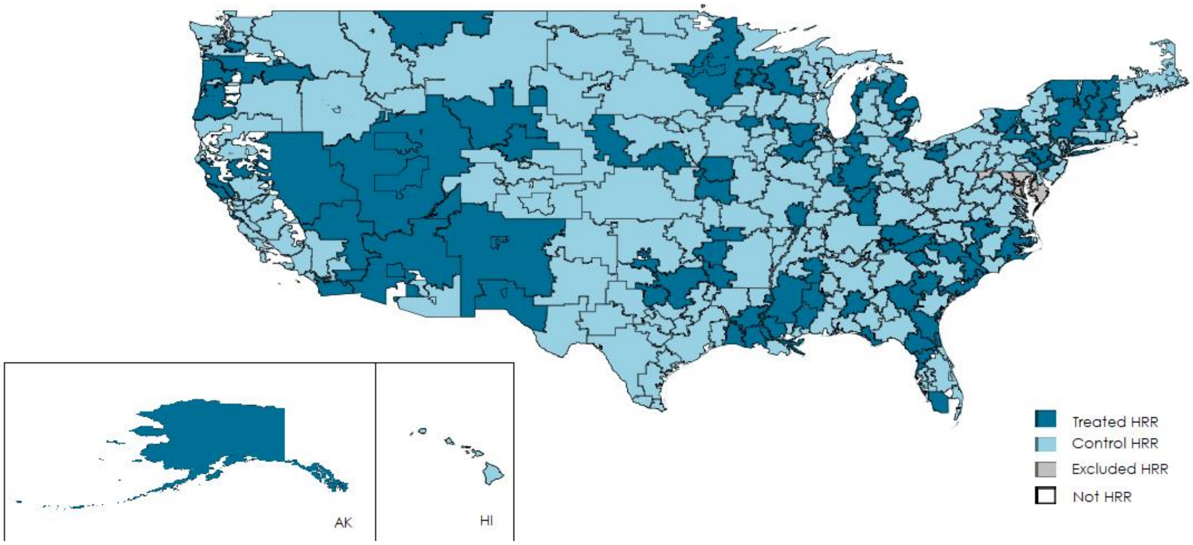

© 2022 Ji Y et al. *JAMA Health Forum.*

eTable 1. Sample Restrictions			
	**Observations**	**Number Lost**	**Percent Lost**
**Starting Total**	327,192		
**HRR Exclusions**	313,669	13,523	4.13
US territories	323,715	3,477	1.06
Maryland HRRs	313,669	10,046	3.10
**Patient Exclusions**	267,109	46,560	14.84
AKI	313,669	-	0.00
Under 18 years old	312,935	734	0.23
Kidney Transplant	312,760	175	0.06
Medicare Part B	312,343	417	0.13
HMO	310,905	1,438	0.46
Lives Outside the US	310,755	150	0.05
On Hospice	304,956	5,799	1.87
Has Dementia	286,548	18,408	6.04
SNF	267,109	19,439	6.78
**Additional Exclusions**	18,621	248,488	93.03
Not a New Patient	55,676	211,433	79.16
Under 66 years old	23,630	32,046	57.56
Started treatment after Oct. 3	18,621	5,009	21.20
**Final Sample**	18,621	308,571	94.31

Notes: Table reports the number of patients who received dialysis and had Medicare as the primary payer. The left-hand column lists the restrictions. The categories in bold give the total for the sub-categories below. HRR and Patient exclusions were determined by the CMS model. Additional exclusions were determined by our team.

© 2022 Ji Y et al. *JAMA Health Forum.*

eTable 2. Robustness to Alternative Controls (2021)

	Mean (SD) Value in Control HRRs		Mean Difference (95% CI) Between Treatment and Control HRRs						P-Value
	(1)		(2)						(3)
Panel A: % Any Home Dialysis in First 90 Days									
No weights	21.74	(10.66)	0.63	(	-1.46	to	2.72	)	0.56
No lagged outcome	20.60	(7.77)	-0.207	(	-2.03	to	1.61	)	0.83
No demographic or health controls	20.60	(7.77)	0.043	(	-1.46	to	1.54	)	0.96
No lagged outcome, demographic or health controls	20.60	(7.77)	0.25	(	-1.70	to	2.20	)	0.80
No weights, lagged outcome, demographic or health controls	21.74	(10.66)	0.004	(	-2.35	to	2.35	)	1.00
Panel B: % Weeks Receiving Any Home Dialysis in First 90 Days									
No weights	17.57	(9.73)	0.36	(	-1.58	to	2.31	)	0.72
No lagged outcome	16.67	(6.77)	-0.33	(	-1.99	to	1.33	)	0.70
No demographic or health controls	16.67	(6.77)	-0.241	(	-1.56	to	1.08	)	0.72
No lagged outcome, demographic or health controls	16.67	(6.77)	-0.285	(	-1.96	to	1.39	)	0.74
No weights, lagged outcome, demographic or health controls	17.57	(9.73)	-0.526	(	-2.70	to	1.65	)	0.64
Panel C: % Dailysis Sessions at Home in First 90 Days									
No weights	18.29	(9.73)	0.48	(	-1.44	to	2.39	)	0.63
No lagged outcome	17.23	(6.81)	-0.23	(	-1.83	to	1.37	)	0.78
No demographic or health controls	17.23	(6.81)	-0.014	(	-1.33	to	1.31	)	0.99
No lagged outcome, demographic or health controls	17.23	(6.81)	-0.002	(	-1.70	to	1.69	)	P>0.99
No weights, lagged outcome, demographic or health controls	18.29	(9.73)	-0.272	(	-2.47	to	1.92	)	0.81
Panel D: Dialysis Rate per Capita									
No weights	0.01	(0.005)	-0.00009	(	-0.0003	to	0.0002	)	0.50
No lagged outcome	0.01	(0.005)	-0.00001	(	-0.0008	to	0.0008	)	0.98

© 2022 Ji Y et al. *JAMA Health Forum.*

No demographic or health controls	0.01	(0.005)	-0.00009	(	-0.0003	to	0.0002	)	0.48
No lagged outcome, demographic or health controls	0.01	(0.005)	-0.00045	(	-0.0017	to	0.0008	)	0.47
No weights, lagged outcome, demographic or health controls	0.01	(0.005)	0.0001	(	-0.001	to	0.001	)	0.93
Panel E: Total Number of Dialysis Patients									
No weights	979	(1260)	10.78	(	-11.30	to	32.85	)	0.34
No lagged outcome	2388	(2521)	-117	(	-781	to	546	)	0.73
No demographic or health controls	2388	(2521)	28	(	-23	to	78	)	0.29
No lagged outcome, demographic or health controls	2388	(2521)	-371	(	-1329	to	588	)	0.45
No weights, lagged outcome, demographic or health controls	979	(1260)	59.13	(	-214.38	to	332.63	)	0.68
Panel F: Pre-Dialysis Elixhauser Index									
No weights	6.06	(0.96)	0.00	(	0.00	to	0.00	)	0.61
No lagged outcome	5.96	(0.75)	0.00	(	0.00	to	0.00	)	0.61
No demographic or health controls	5.96	(0.75)	-0.01	(	-0.17	to	0.15	)	0.91
No lagged outcome, demographic or health controls	5.96	(0.75)	0.06	(	-0.12	to	0.24	)	0.53
No weights, lagged outcome, demographic or health controls	6.06	(0.96)	-0.08	(	-0.29	to	0.13	)	0.46
Panel G: % Any Home Dialysis in First 90 Days in 2020									
No weights	21.40	(11.37)	-2.41	(	-4.49	to	-0.32	)	0.03
No lagged outcome	20.00	(8.55)	-1.25	(	-2.99	to	0.50	)	0.17
No demographic or health controls	20.00	(8.55)	-1.02	(	-2.68	to	0.64	)	0.23
No lagged outcome, demographic, or health controls	20.00	(8.55)	-1.04	(	-3.12	to	1.04	)	0.33
No weights, lagged outcome, demographic or health controls	21.40	(11.37)	-2.12	(	-4.70	to	0.46	)	0.11

Notes: Table replicates Table 3, except with different HRR-level covariates or weights. Each of the panels show how the outcome is affected by varying the specification. The first specification assigns equal weights to HRRs; the second specification controls for HRR-level patient demographics and pre-dialysis health, but not for lagged outcome variable; the third specification controls for lagged outcome variable but not for HRR-level patient demographics or pre-dialysis health; the fourth specification does not control for either; the last specification does not control for either and assigns equal weights to HRRs.

© 2022 Ji Y et al. *JAMA Health Forum.*

eTable 3. Robustness to Alternative Controls, Heterogeneity by Patient and Facility Characteristics (2021)

	Mean (SD) Value in Control HRRs		Mean Difference (95% CI) Between Treatment and Control HRRs						P-Value	P-Value of Difference
	(1)		(2)						(3)	(4)
Panel A: No weights										
Heterogeneity by Patient Characteristics										
Non-Medicaid	24.80	11.76	-0.06	(	-2.35	to	2.22	)	0.96	0.50
Medicaid	8.86	12.67	1.18	(	-1.86	to	4.23	)	0.45	
Non-White	15.60	16.57	-0.61	(	-4.59	to	3.38	)	0.77	0.52
White	24.03	11.76	0.80	(	-1.47	to	3.07	)	0.49	
Rural	13.50	13.43	0.95	(	-2.63	to	4.53	)	0.60	0.83
Urban	24.58	13.17	0.46	(	-2.54	to	3.45	)	0.76	
Non-Disabled	23.24	11.50	0.54	(	-1.61	to	2.70	)	0.62	0.02
Disabled	14.10	15.10	5.45	(	1.82	to	9.09	)	0.01	
Above Median Distance to Nearest Facility	22.43	12.98	1.33	(	-1.33	to	3.99	)	0.33	0.18
Below Median Distance to Nearest Facility	19.50	19.31	-1.89	(	-5.92	to	2.15	)	0.36	
Heterogeneity by Facility Characteristics										
Non-Profit	16.75	20.03	0.66	(	-5.90	to	7.23	)	0.84	0.47
For-Profit	22.01	11.90	-1.90	(	-4.44	to	0.65	)	0.14	
Non-Chains	20.28	20.02	0.13	(	-4.45	to	4.72	)	0.96	0.66
Chains (Fresinius or DaVita)	22.42	13.58	-1.11	(	-3.98	to	1.77	)	0.45	
Above Median Volume	22.77	14.86	0.16	(	-2.81	to	3.14	)	0.91	0.47
Below Median Volume	21.52	20.68	-1.67	(	-5.33	to	1.99	)	0.37	
Panel B: No lagged outcome										
Heterogeneity by Patient Characteristics										
Non-Medicaid	23.92	8.36	-0.57	(	-2.45	to	1.32	)	0.56	0.13
Medicaid	9.45	9.34	1.63	(	-0.71	to	3.97	)	0.17	
Non-White	15.05	11.10	-0.65	(	-3.50	to	2.21	)	0.66	0.63
White	23.28	8.89	0.13	(	-1.88	to	2.13	)	0.90	
Rural	14.83	12.60	0.26	(	-2.97	to	3.49	)	0.87	0.94
Urban	22.45	9.94	0.11	(	-2.36	to	2.58	)	0.93	
Non-Disabled	21.75	8.60	-0.60	(	-2.43	to	1.24	)	0.52	0.06
Disabled	14.96	10.86	2.35	(	-0.40	to	5.10	)	0.09	
Above Median Distance to Nearest Facility	21.89	10.96	-0.54	(	-2.77	to	1.68	)	0.63	0.98
Below Median Distance to Nearest Facility	18.61	11.63	-0.58	(	-3.17	to	2.02	)	0.66	
Heterogeneity by Facility Characteristics										
Non-Profit	16.42	19.52	1.01	(	-6.28	to	8.30	)	0.79	0.74
For-Profit	20.69	8.72	-0.29	(	-2.81	to	2.23	)	0.82	

© 2022 Ji Y et al. *JAMA Health Forum.*

Non-Chains	19.54	15.42	-0.27	(	-4.15	to	3.60	)	0.89	0.53
Chains (Fresinius or DaVita)	20.86	9.55	-1.69	(	-3.98	to	0.59	)	0.15	
Above Median Volume	20.67	11.27	0.99	(	-1.41	to	3.39	)	0.42	0.42
Below Median Volume	21.64	15.32	-0.76	(	-4.15	to	2.62	)	0.66	
Panel C: No demographic or health controls										
Heterogeneity by Patient Characteristics										
Non-Medicaid	23.92	8.36	-0.60	(	-2.42	to	1.23	)	0.52	0.06
Medicaid	9.45	9.34	2.19	(	-0.10	to	4.48	)	0.06	
Non-White	15.05	11.10	0.09	(	-2.70	to	2.89	)	0.95	0.88
White	23.28	8.89	-0.15	(	-1.99	to	1.70	)	0.88	
Rural	14.83	12.60	0.36	(	-3.23	to	3.95	)	0.84	0.55
Urban	22.45	9.94	-0.83	(	-3.08	to	1.42	)	0.47	
Non-Disabled	21.75	8.60	-0.16	(	-1.85	to	1.53	)	0.85	0.05
Disabled	14.96	10.86	2.86	(	0.07	to	5.66	)	0.04	
Above Median Distance to Nearest Facility	21.89	10.96	0.47	(	-1.99	to	2.93	)	0.71	0.80
Below Median Distance to Nearest Facility	18.61	11.63	0.93	(	-1.82	to	3.67	)	0.51	
Heterogeneity by Facility Characteristics										
Non-Profit	16.42	19.52	4.78	(	-2.11	to	11.66	)	0.17	0.16
For-Profit	20.69	8.72	-0.27	(	-2.42	to	1.87	)	0.80	
Non-Chains	19.54	15.42	-1.08	(	-4.44	to	2.27	)	0.53	0.57
Chains (Fresinius or DaVita)	20.86	9.55	0.11	(	-2.08	to	2.31	)	0.92	
Above Median Volume	20.67	11.27	0.57	(	-1.80	to	2.94	)	0.64	0.12
Below Median Volume	21.64	15.32	-2.81	(	-6.08	to	0.46	)	0.09	
Panel D: No lagged outcome, demographic or health controls										
Heterogeneity by Patient Characteristics										
Non-Medicaid	23.92	8.36	-0.51	(	-2.53	to	1.51	)	0.62	0.05
Medicaid	9.45	9.34	2.38	(	0.04	to	4.72	)	0.05	
Non-White	15.05	11.10	0.01	(	-2.86	to	2.88	)	0.99	0.74
White	23.28	8.89	0.53	(	-1.54	to	2.60	)	0.61	
Rural	14.83	12.60	0.39	(	-3.32	to	4.09	)	0.84	0.67
Urban	22.45	9.94	-0.51	(	-3.24	to	2.21	)	0.71	
Non-Disabled	21.75	8.60	0.02	(	-1.99	to	2.03	)	0.98	0.07
Disabled	14.96	10.86	2.83	(	0.03	to	5.63	)	0.05	
Above Median Distance to Nearest Facility	21.89	10.96	0.81	(	-1.75	to	3.36	)	0.54	0.92
Below Median Distance to Nearest Facility	18.61	11.63	0.64	(	-2.16	to	3.43	)	0.66	
Heterogeneity by Facility Characteristics										
Non-Profit	16.42	19.52	3.76	(	-4.20	to	11.71	)	0.35	0.47
For-Profit	20.69	8.72	0.74	(	-1.93	to	3.42	)	0.59	
Non-Chains	19.54	15.42	-1.33	(	-5.27	to	2.61	)	0.51	0.43
Chains (Fresinius or DaVita)	20.86	9.55	0.51	(	-1.93	to	2.95	)	0.68	
Above Median Volume	20.67	11.27	1.04	(	-1.69	to	3.77	)	0.46	0.22
Below Median Volume	21.64	15.32	-2.18	(	-6.13	to	1.76	)	0.28	
Panel E: No weights, lagged outcome, demographic or health controls										
Heterogeneity by Patient Characteristics										
Non-Medicaid	24.80	11.76	0.13	(	-2.60	to	2.86	)	0.93	0.45

© 2022 Ji Y et al. *JAMA Health Forum.*

Medicaid	8.86	12.67	1.66	(	-1.41	to	4.74	)	0.29	
Non-White	15.60	16.57	-1.01	(	-5.26	to	3.23	)	0.64	0.34
White	24.03	11.76	1.23	(	-1.36	to	3.82	)	0.35	
Rural	13.50	13.43	1.63	(	-2.51	to	5.77	)	0.44	0.24
Urban	24.58	13.17	-1.43	(	-5.02	to	2.15	)	0.43	
Non-Disabled	23.24	11.50	-0.07	(	-2.72	to	2.59	)	0.96	0.01
Disabled	14.10	15.10	5.48	(	1.63	to	9.32	)	0.01	
Above Median Distance to Nearest Facility	22.43	12.98	1.65	(	-1.40	to	4.71	)	0.29	0.36
Below Median Distance to Nearest Facility	19.50	19.31	-0.73	(	-5.30	to	3.85	)	0.76	
Heterogeneity by Facility Characteristics										
Non-Profit	16.75	20.03	2.21	(	-5.41	to	9.82	)	0.57	0.69
For-Profit	22.01	11.90	0.46	(	-3.51	to	4.42	)	0.82	
Non-Chains	20.28	20.02	-1.36	(	-6.58	to	3.85	)	0.61	0.68
Chains (Fresinius or DaVita)	22.42	13.58	-0.04	(	-3.65	to	3.58	)	0.98	
Above Median Volume	22.77	14.86	-0.46	(	-4.09	to	3.17	)	0.80	0.60
Below Median Volume	21.52	20.68	-2.25	(	-7.32	to	2.82	)	0.38	

Notes: Table replicates Table 4, except with different HRR-level covariates or weights. Each of the panels show a different specification. The first specification assigns equal weights to HRRs; the second specification controls for HRR-level patient demographics and pre-dialysis health, but not for lagged outcome variable; the third specification controls for lagged outcome variable but not for HRR-level patient demographics or pre-dialysis health; the fourth specification does not control for either; the last specification does not control for either and assigns equal weights to HRRs.

eTable 4. Impact of ETC During First Year of the Program (2021), 30-Day Outcomes

	Mean (SD) Value in Control HRRs		Mean Difference (95% CI) Between Treatment and Control HRRs						P-Value
	(1)		(2)						(3)
% Any Home Dialysis in First 30 Days	18.33	(10.11)	0.272	(	-1.21	to	1.76	)	0.72
% Weeks Receiving Any Home Dialysis in First 30 Days	17.53	(9.68)	0.165	(	-1.25	to	1.58	)	0.82
% Dialysis Sessions at Home in First 30 Days	17.18	(9.56)	0.167	(	-1.22	to	1.55	)	0.82
% Any Home Dialysis in First 30 Days in 2020	18.39	(10.40)	-1.528	(	-3.01	to	-0.04	)	0.05

Notes: Table replicates Table 3 Panel (a), except that all outcomes are defined during the first 30 days of dialysis, rather than the first 90 days.

eTable 5. Impact of ETC on Home Dialysis and Self Dialysis (2021)					
	Mean (SD) Value in Control HRRs		Mean Difference (95% CI) Between Treatment and Control HRRs		P-Value
	(1)		(2)		(3)
% Any Home Dialysis in First 90 Days	20.60	(7.77)	0.12	(-1.42 to 1.65)	0.89
% Any Self Dialysis in First 90 Days	0.02	(0.08)	0.02	(-0.04 to 0.08)	0.50
% Any Home Dialysis or Self Dialysis in First 90 Days	20.60	(7.77)	0.12	(-1.41 to 1.66)	0.88

Notes: The first row replicates the first row in Table 3; subsequent rows replicate the same analysis for additional outcomes.

© 2022 Ji Y et al. *JAMA Health Forum.*

eTable 6. Impact of ETC During First Year of the Program (2021)^

	Mean (SD) Value in Control HRRs		Mean Difference (95% CI) Between Treatment and Control HRRs						P-Value
	(1)		(2)						(3)
% All ETC Eligible Patients with Any Home Dialysis in 2021*	17.60	(5.50)	0.22	(	-0.40	to	0.84	)	0.48
% Existing ETC Eligible Patients with Any Home Dialysis during 2021	16.17	(5.34)	0.26	(	-0.36	to	0.88	)	0.41

Notes: Table reports HRR-level average characteristics of ETC-eligible patients. Column (1) reports the means for the control HRRs. Column (2) reports the coefficient on the treatment indicator from estimating equation (1), which is an HRR-level regression of the outcome variable on the treatment indicator, controlling for strata fixed effects, lagged outcome from three years prior, and HRR-level averages of patient demographic and baseline health. Baseline health is measured by Elixhauser comorbidities, which we constructed using inpatient and outpatient claims from 2020 and 2021. The regression is weighted by the average number of patients in our sample in 2018 and 2019. We report heteroskedasticity robust standard errors. Existing ETC Eligible Patients refer to patients who had at least one session of dialysis during the last 10 days of December 2020.
* Includes all Medicare ESRD patients who met the ETC eligibility requirements.
^ This table was not pre-specified.

© 2022 Ji Y et al. *JAMA Health Forum.*

eTable 7. Impact of ETC During First Year of the Program Controlling for Benchmark Year Achievement (2021)^

		Mean (SD) Value in Control HRRs				Mean Difference (95% CI) Between Treatment and Control HRRs						P-Value
		(1)				(2)						(3)
% Any Home Dialysis in First 90 Days		20.58	(	15.31	)							
	Treatment					2.17	(	-1.22	to	5.55	)	0.22
	Quintile 2					0.08	(	0.05	to	11.37	)	P<0.001
	Quintile 3					0.16	(	0.14	to	18.54	)	P<0.001
	Quintile 4					0.20	(	0.18	to	22.61	)	P<0.001
	Quintile 5					0.34	(	0.30	to	37.58	)	P<0.001
	Treatment*Q2					-1.27	(	-6.36	to	3.81	)	0.63
	Treatment*Q3					-1.87	(	-5.99	to	2.24	)	0.38
	Treatment*Q4					-2.17	(	-6.53	to	2.18	)	0.33
	Treatment *Q5					-5.35	(	-11.50	to	0.80	)	0.09
% Weeks Receiving Any Home Dialysis in First 90 Days		16.61	(	14.32	)							
	Treatment					0.92	(	-1.45	to	3.28	)	0.45
	Quintile 2					7.68	(	5.27	to	10.09	)	P<0.001
	Quintile 3					14.84	(	12.97	to	16.71	)	P<0.001
	Quintile 4					18.15	(	16.12	to	20.18	)	P<0.001
	Quintile 5					31.51	(	27.96	to	35.06	)	P<0.001
	Treatment*Q2					-0.22	(	-4.88	to	4.45	)	0.93
	Treatment*Q3					-0.21	(	-3.41	to	2.99	)	0.90
	Treatment*Q4					-1.54	(	-5.03	to	1.95	)	0.39
	Treatment *Q5					-4.53	(	-10.23	to	1.16	)	0.12
% Dialysis Sessions at Home in First 90 Days		17.19	(	14.24	)							
	Treatment					1.22	(	-1.17	to	3.61	)	0.32
	Quintile 2					7.86	(	5.40	to	10.33	)	P<0.001
	Quintile 3					14.65	(	12.79	to	16.51	)	P<0.001
	Quintile 4					18.36	(	16.30	to	20.42	)	P<0.001
	Quintile 5					31.15	(	27.60	to	34.70	)	P<0.001
	Treatment*Q2					-0.58	(	-4.86	to	3.70	)	0.80
	Treatment*Q3					-0.35	(	-3.52	to	2.81	)	0.83
	Treatment*Q4					-1.67	(	-5.12	to	1.78	)	0.35
	Treatment *Q5					-4.45	(	-9.98	to	1.08	)	0.12

© 2022 Ji Y et al. *JAMA Health Forum.*

Pre-Dialysis Elixhauser Index	5.96	(	1.25	)							
Treatment					0.36	(	-0.14	to	0.87	)	0.17
Quintile 2					0.01	(	-0.43	to	0.44	)	0.97
Quintile 3					0.03	(	-0.29	to	0.35	)	0.87
Quintile 4					-0.13	(	-0.46	to	0.20	)	0.44
Quintile 5					-0.56	(	-0.93	to	-0.20	)	0.003
Treatment*Q2					-0.62	(	-1.41	to	0.18	)	0.13
Treatment*Q3					-0.25	(	-0.81	to	0.31	)	0.38
Treatment*Q4					-0.52	(	-1.08	to	0.03	)	0.07
Treatment *Q5					-0.24	(	-0.88	to	0.39	)	0.46
% Any Home Dialysis in First 90 Days in 2020	0.20	(	0.16	)							
Treatment					0.01	(	-0.03	to	0.06	)	0.52
Quintile 2					0.07	(	0.04	to	0.10	)	P<0.001
Quintile 3					0.14	(	0.12	to	0.16	)	P<0.001
Quintile 4					0.19	(	0.16	to	0.21	)	P<0.001
Quintile 5					0.33	(	0.29	to	0.38	)	P<0.001
Treatment*Q2					0.02	(	-0.05	to	0.08	)	0.60
Treatment*Q3					-0.02	(	-0.07	to	0.03	)	0.40
Treatment*Q4					-0.01	(	-0.06	to	0.04	)	0.66
Treatment *Q5					-0.07	(	-0.15	to	0.00	)	0.05

Notes: Table reports facility group-level average characteristics of patients in our baseline sample. Column (1) reports the means for the control facility groups. Column (2) reports the coefficients from estimating a facility group-level regression of the outcome variable on the treatment indicator, indicators for achievement quintiles in the benchmark year, the interaction between the treatment indicator and indicators for the achievement quintiles, controlling for strata fixed effects, lagged outcome from three years prior, and facility group-level averages of patient demographic and baseline health. Achievement quintiles were calculated using all facilities during the benchmark year (July 2019-June 2020) and all ETC-eligible patients. Facility groups with lower achievement quintiles in benchmark years may have stronger incentives to improve in the performance year. The regression is weighted by the number of eligible patients in each facility group in 2020. We report 95% confidence intervals based on heteroskedasticity robust standard errors.

^This table was not pre-specified.

© 2022 Ji Y et al. *JAMA Health Forum.*

eAppendix

Pre-Analysis Plan for

Financial Incentives to Facilities and Clinicians Treating Patients With End Stage Renal Disease and Use of Home Dialysis: Evidence from the First Year of a US Nationwide Randomized Clinical Trial.

Date: July 30, 2021

Liran Einav[1], Amy Finkelstein[2], Yunan Ji[3], and Neale Mahoney[1]

Abstract

This document pre-specifies our planned analyses of the first year of a nationwide randomized-controlled trial of end stage renal disease (ESRD) treatment choice. This mandatory-participation program was designed by the Centers for Medicare and Medicaid Services and randomization was conducted at the hospital referral region (HRR) level. 95 HRRs were assigned to the treatment group beginning in January 2021. We will study the impact of this program in the first year on treatment modality choice for ESRD and explore heterogeneity in impact across patients and providers.

DEPARTMENT(S):

1. Department of Economics, Stanford University
2. Department of Economics, Massachusetts Institute of Technology and Abdul Latif Jameel Poverty Action Lab, Massachusetts Institute of Technology
3. Graduate School of Arts and Sciences, Harvard University

© 2022 Ji Y et al. *JAMA Health Forum.*

1. Introduction

Treatment for end stage renal disease (ESRD) requires a large amount of health care resources: the ESRD population accounts for 7% of Medicare fee-for-service spending despite making up less than 1% of total Medicare enrollment (USRDS 2020). The most common treatment for ESRD is dialysis, for which there are two options: home dialysis (which usually takes the form of peritoneal dialysis) and facility dialysis (which is usually hemodialysis). Home dialysis is often associated with lower spending and better outcomes than facility-based dialysis (GAO 2015). However, only 12% of patients receive dialysis at home in the US, significantly below that of other developed countries (USRDS 2020).

We propose to study the ESRD Treatment Choice (ETC) program, a nationwide randomized-controlled trial (RCT) of ESRD treatment that was designed by the Centers for Medicare and Medicaid Services (CMS) to encourage greater use of home dialysis. The RCT creates payment adjustments for ESRD facilities and clinicians based on the rates of home dialysis and transplantation. The RCT was first announced in July 2019 for a start date of January 1, 2020 which was later delayed to January 1, 2021. In September 2020, 30% of hospital referral regions (HRRs) were assigned to treatment through a stratified randomization procedure. The RCT is scheduled to last until June 2027.

This analysis plan is for the first year of the program, which covers calendar year 2021.[1] We will examine the impact of payment incentives for home dialysis on home dialysis use among ESRD patients. We examine both the average impacts, as well as heterogeneity across provider types and patient characteristics.

2. Experimental Design

The ETC program is a nation-wide, HRR-level, mandatory RCT. All Medicare-certified ESRD facilities and Medicare-enrolled managing clinicians in the selected HRRs are required to participate. In this section, we summarize the details of the program and describe our study sample.

2.1 Program Incentives

The ETC model makes two types of payment adjustments for facilities and managing clinicians, an adjustment to the reimbursement rate for home dialysis, and a performance adjustment. Although they are implemented together and we analyze them jointly, we expect the performance payment adjustment to have a bigger effect given the greater downside and upside risks.

The first adjustment, **Home Dialysis Payment Adjustment (HDPA)**, raises the reimbursement rate for home dialysis for the first three years of the program; the amount of increase is 3% in 2021, and reduces to 2% in 2022, and 1% in 2023 (CMS 2020).

The second adjustment, **Performance Payment Adjustment (PPA)**, is an increase or decrease in the reimbursement rate based on the home dialysis rate and the transplant rate attributable to the participating facility or clinician. The PPA, unlike the HDPA, has separate measurement years (during which CMS assess the performance of each participant) and performance payment adjustment periods

[1] As of July 2021, there is a proposed modification of the program starting in year 2 (2022) that provides additional incentives for improving home dialysis and transplant rates for low-income individuals. https://www.cms.gov/newsroom/press-releases/cms-proposes-changes-reduce-health-care-disparities-among-patients-chronic-kidney-disease-and-end

© 2022 Ji Y et al. *JAMA Health Forum.*

(during which payments are adjusted based on the performance in the corresponding measurement year). The first measurement year is the calendar year 2021, which affects payments made to providers in the period between July and December of 2022.[2] The magnitude of the adjustment depends on the modality performance score (MPS). Specifically, MPS includes two assessment measures –home dialysis rate (including both home dialysis and in-facility self-dialysis)[3], and transplant rate (including both transplant waitlisting and living donor transplants). The transplant waitlisting rate is risk-adjusted based on age; home dialysis rates and living donor rates are not risk adjusted.[4] For each of the two measures, each facility or clinician receives an achievement score and an improvement score. Specifically, participants earn between 0 and 2 points (in increments of 0.5) in achievement score based on how their home dialysis and transplant rates in the measurement year compare with those in control areas in the benchmark year. Participants earn between 0 and 1.5 points (in increments of 0.5) in improvement score based on how their home dialysis and transplant rates in the measurement year compare with their own performance during the benchmark year. For both measures, the benchmark year is defined as the 12-month period that starts 18 months before each measurement year.

The MPS is then computed as follows:

Modality Performance Score

= 2(Higher of home dialysis achievement or improvement score)*

+ (Higher of transplant achievement or improvement score)

Providers with an MPS between 2 (exclusive) and 3.5 (inclusive) do not receive a payment adjustment; providers with an MPS below 2 (inclusive) receive a negative payment adjustment and providers with an MPS above 3.5 (exclusive) receive a positive payment adjustment. The magnitude of the adjustment increases over time, from -5% to +4% in the first year to -10% to +8% in the last year (CMS 2020).

In our analysis, we will focus on home dialysis rates rather than transplant rates for several reasons. First, the formula for computing MPS gives home dialysis double the weight as transplants, hence there would be stronger incentives for improving home dialysis. Second, because the living donor transplant rate is difficult to change given the severe shortage in kidney supply (McCormick et al. 2018), we expect most effects on transplant rates to come from increased transplant waitlisting, which we are unable to observe in our Medicare claims data. Furthermore, based on our power calculation using the Medicare claims, we are unable to detect meaningful changes in (realized) transplant rates.[5] Finally, we think transplant waitlisting is unlikely to affect home dialysis, as both can be achieved independently; since we

---

[2] The second measurement year is July 1, 2021 to June 30, 2022 (which overlaps with half of the first measurement year), which affects the payments between January 1, 2023 and June 30, 2023. The last (10th) measurement year is July 1, 2025 to June 30, 2026, which affects the payments between January 1, 2027 and June 30, 2027.

[3] Self-dialysis is counted as ½ of home dialysis for the purpose of computing MPS. The rationale for including self-dialysis is that it provides training for patients to eventually transition to home dialysis. (CMS 2020) Based on our analysis of the 2016 Medicare claims data, only 0.04% of dialysis claims are “self-dialysis.”

[4] Transplant waitlist rate is risk adjusted based on beneficiary age at the end of each month. Specifically, there are separate risk coefficients for beneficiaries aged 19-55, 56-70, and 71-74. The risk adjustment accounts for the relative percentage of beneficiaries attributed to each provider relative to the national age distribution of beneficiaries. (CMS 2020)

[5] We estimate that the minimum detectable effect size for transplant rate is about 80% of the control mean based on our power calculation.

© 2022 Ji Y et al. *JAMA Health Forum.*

expect the change in actual transplant rates to be moderate, we do not expect it to have a first-order impact on the number of patients undergoing dialysis. Nonetheless, we will examine the total number of dialysis patients as an outcome measure to detect any extensive margin response.

2.2 Nature of Incentives

Both HDPA and PPA provide financial incentives for increasing home dialysis, and these incentives can have both a substitution effect - in which providers steer ESRD patients toward home dialysis - and an income effect which can affect patients in all treatment modalities. First, ETC raises payments for home dialysis, rewards providers for increasing home dialysis rates and penalizes providers who do not utilize home dialysis adequately, which can cause providers to dialyze fewer patients in facilities and more at home. We therefore expect this substitution effect to increase the number of patients in home dialysis and decrease the number of patients in facility dialysis. Second, increased payments in HDPA and (conditional on performance) PPA provide additional income to the providers, which can have an impact on both the quantity and quality of all the services they provide, regardless of the treatment modality. For example, the additional income could affect the number of dialysis stations the facility acquires, the number of clinicians it hires, and the amount of time devoted to each patient, among many other things. Conversely, reduced revenue from poor performance on PPA can also have an impact on all patients through similar channels. We have no a priori hypothesis of the sign of this income effect on home dialysis rates.

The computation of improvement scores, which compares one's performance during the measurement year with that in a benchmark year that is 18 months prior, could result in ratchet effects. With the exception of the first calendar year (2021) of the program, all subsequent years use at least part of a previous performance measurement year as a benchmark; such design can dampen the incentive to improve quickly since doing so will make the target harder to meet in later years. In the period between the announcement of the program in 2019 and its final implementation in 2021, there was an additional incentive for providers to lower their home dialysis and transplant rates as they can do so without incurring any penalty while achieving a favorable benchmark rate in anticipation of the program launch. To examine such effects, we analyze home dialysis rate in 2020 as one of our outcomes.

Finally, providers have the incentive to "cream skim" or attract patients who are more suitable for home dialysis (as opposed to facility dialysis) to improve their home dialysis rates. These incentives are exacerbated by the fact that currently there is no risk adjustment mechanism when computing home dialysis rates.

2.3 Randomization

Any HRR with 20% or more zip codes in Maryland are excluded from the randomization and directly assigned to the program. 30% of the remaining HRRs are randomly selected to participate in the program, through a stratified randomization by Census-defined regions (Northeast, South, Midwest, and West) (CMS 2020).

2.4 Program Eligibility and Study Sample

Our baseline sample includes all Medicare-certified ESRD facilities and Medicare-enrolled managing clinicians located in HRRs that are eligible for randomization. Following the program rules (CMS 2020), we make the following sample restrictions. In 2016[6], a total of 394,323 Medicare beneficiaries with Medicare as their primary insurance received dialysis. For each group of restrictions, we report the number of beneficiaries excluded from our sample.

We begin by imposing three restrictions that the ETC program imposes:

- HRR exclusion criteria (16,567 beneficiaries in 4 HRRs excluded in total based on these criteria):
    - US territories are excluded.
    - Any HRR with 20% or more zip codes in Maryland are excluded from the randomization.[7]
- Facility and clinician exclusion criteria[8]
    - Facilities and clinicians with fewer than 11 attributed beneficiaries are excluded from the performance payment adjustment.
- Patient exclusion criteria: an ESRD beneficiary is excluded if at any point during the first 90 days of dialysis, the beneficiary[9] (104,294beneficiaries excluded in total):[10]
    - Is receiving dialysis only for an acute kidney injury (AKI),
    - Is younger than 18 years of age before the first day of the month of the claim service date,
    - Received a kidney transplant within 12 months prior to the start of dialysis and does not have a kidney transplant failure code,
    - Is not enrolled in Medicare Part B[*],
    - Is enrolled in Medicare Advantage, a cost plan, or other Medicare managed care plan[*],
    - Does not reside in the US[*],
    - Has elected hospice[*],
    - Has a diagnosis of dementia at any point during the month of the claim service date or the preceding 12 months, identified using CMS-HCC[*],
    - Is residing in or receiving dialysis in a skilled nursing facility (SNF) [*].
- In addition to the sample restrictions imposed by the ETC program, we make several additional sample restrictions in our analysis (254,758beneficiaries excluded in total):
    - Patient must be new to dialysis, defined as not having received dialysis for at least 12 months prior to their first observed dialysis claim, (Excludes 211,258 beneficiaries)
    - Patient must be at least 66 years old on the first month of dialysis, this ensures that we observe at least 12 months of Medicare claims prior to their first dialysis, (Excludes 38,146 beneficiaries)

---

[6] Our analysis sample will be based on patients in 2021, but for purposes of the pre-analysis plan, we have implemented our sample definitions on the 2016 patients.

[7] Maryland is assigned by CMS to participate in ETC. However, since the assignment is not based on randomization, we exclude Maryland from our analysis.

[8] Since we only have a 20% Medicare carrier file at the moment, we do not have an estimate for how many patients are excluded from these criteria.

[9] The restrictions marked with [*] only apply to the calculation of PPA based on the official rules. (CMS 2020) However, since HDPA and PPA are introduced at the same time and we expect most of the effect to come from PPA due to the stronger financial incentives, we restrict our baseline sample to the set of patients meeting the requirements for both adjustments. We may explore heterogeneity by looking separately at patients included in HDPA but not PPA.

[10] The official rule applies on a month-by-month basis to all dialysis patient-months. However, since we are only looking at 90-day outcomes for new patients we modify these to apply to the first 90 days of dialysis treatment.

© 2022 Ji Y et al. *JAMA Health Forum.*

- Patient begins dialysis at least 90 days before the end of 2021 (to allow us to construct 90-day outcomes). (Excludes 5,354 beneficiaries)

We restrict to new dialysis patients for purposes of improving statistical power; we suspect the main impact of the program to be on choice of dialysis modality for new patients, rather than the moving of existing patients. The other sample restrictions are designed to ensure that we observe the patient's baseline health as well as at least 90 days of outcomes from the start of dialysis.

After accounting for the appropriate exclusions, 302 HRRs were eligible for randomization, of which 91 HRRs were assigned to treatment. Using the 2016 data, the analysis sample includes 18,704 beneficiaries, out of which 5,773 are in treatment.

3. Proposed Analyses

3.1 Baseline Specification

This analysis plan is for the first year of the program, which begins on Jan 1, 2021, and ends on Dec 31, 2021. We will conduct our baseline analysis using the 100% Medicare claims data aggregated to the HRR level. Our main regression specification examines the direct impact of the ETC program on rates of home dialysis rates.

Let j denote HRRs and let t denote calendar years. Let y_j denote the average outcome in HRR j in the first year of the program. Let z_j be a binary variable that takes on a value of 1 for the HRRs assigned to treatment and 0 for the HRRs assigned to control. Our baseline reduced form specification is given by

$$y_j = \beta_0 + \beta_1 z_j + \beta_{2,s(j)} + \beta_3 y_{pre,j} + X_j + \epsilon_j \qquad (1)$$

The coefficient of interest is β_1. $\beta_{2,s(j)}$ denotes strata fixed effects; we control for strata fixed effects because, as described above, randomization was performed within strata (specifically, within four census regions). $y_{pre,j}$ denotes lagged outcome from prior to the ETC program, which helps improve statistical power; specifically, we control for outcomes from three years prior to the first program year (i.e. 2018) to avoid anticipatory effects from biasing our estimates, as the ETC program was announced two years prior to its launch. X_j denotes HRR-level covariates, which are also designed to improve power; these consist of average demographics and baseline health among patients in our baseline sample. Specifically, the demographic variables include indicators for fully interacted age-race-sex bins.[11] Baseline health includes separate indicators for the 31 Elixhauser chronic conditions measured in the 12 months prior to dialysis treatment. All measures are averaged at the HRR level. As shown in Appendix Table 1, since the HRRs are heterogeneous in ESRD patient size, we weight this regression by the number of patients in each HRR. We report heteroskedasticity robust standard errors.

3.2 Planned Outcomes

We measure all outcomes using the Medicare fee-for-service claims data.

90-Day Spending and Outcome Measures:

[11] Age is coded in five-year bins (with 85 and over as one group), sex is coded as an indicator for male, and race is coded as white, Black, and other.

© 2022 Ji Y et al. *JAMA Health Forum.*

a) **Percentage of Patients Receiving Any Home Dialysis in First 90 Days.** This is our ***primary*** outcome. It is defined as the percentage of patients in our baseline sample who have any home dialysis claims within the first 90 days since the start of dialysis treatment.
b) **Percentage of Weeks Receiving Any Home Dialysis in First 90 Days.** For each patient, this is the number of weeks the patient received dialysis at home or receiving training for home dialysis within the first 90 days divided by the number of weeks the patient received dialysis in any modality in the first 90 days. For the 13th week since the start of dialysis, we only count the 6 days that fall within the 90 day period and weight it at 6/7 of the other weeks when computing this measure.[12]
c) **Percentage of Dialysis Sessions at Home in First 90 Days.** For each patient, this is the number of dialysis sessions at home divided by the total number of dialysis sessions in the first 90 days, weighting peritoneal dialysis sessions as 3/7 of hemodialysis sessions.[13]

We have decided to focus only on measures of home dialysis use because in our power calculations we have concluded that we lack the power to detect changes in downstream outcomes, including spending, hospitalization, and mortality.[14]

Patient Characteristics and Extensive Margin Outcomes:

We additionally check whether there are changes to the population who receive dialysis over the study period. For example, facilities might have incentives to try to attract patients who are good candidates for home dialysis, although the risk adjustment in the incentives is designed to address this, such effects could affect the interpretation of our estimates of the impact of ETC. Separately, facilities may be incentivized to lower their home dialysis rates during the benchmark year in order to achieve a better performance score during the measurement year.

a) **New Dialysis Patients per Capita.** This is the number of Traditional Medicare patients aged 66 and above who start dialysis in either modality in our baseline sample divided by the number of Traditional Medicare patients aged 66 and above.

[12] We count the share of weeks in home dialysis rather than the share of claims or treatment sessions because hemodialysis and peritoneal dialysis both require sessions each week but differ in the number of sessions required.
[13] We identify sessions by revenue code lines. From Chapter 8 of the Medicare Claims Processing Manual, "effective April 1, 2007, the implementation of ESRD line item billing requires that each dialysis session be billed on a separate line." The 3/7 adjustment reflects the difference in average treatment frequencies between the modalities and is the ratio used in setting Medicare reimbursement rates. See Section 80.4 of the Medicare Claims Processing Manual. Specifically, we count the number dialysis sessions recorded in the revenue file and label sessions as hemodialysis or peritoneal dialysis based on the revenue center code.
[14] To examine the causal impact of home dialysis on downstream outcomes, we could use the random assignment to treatment as an instrument for home dialysis. In this instrumental variable specification, the minimum detectible effect size (MDE) under homoskedasticity with a power of 80% at a significance level of 5% is given by $2.8\frac{\hat{\sigma}}{\sqrt{R_Z^2 n}}$ where $\hat{\sigma}$ is the standard deviation of the outcome in the sample, R_Z^2 is the R-squared of the first stage, and n is the sample size. Compared with the MDE in an OLS setting ($2.8\frac{\hat{\sigma}}{\sqrt{n}}$), the effect size is scaled by $\frac{1}{\sqrt{R_Z^2}}$ under the instrumental variable specification. We have estimated that R_Z^2=0.0017, which makes detecting changes in downstream outcomes challenging. Specifically, for 90-day hospitalizations we have computed a MDE of 41 percentage points (relative to a mean of 22.6%), and for 90-day mortality rate a MDE of 8 percentage points (relative to a mean of 6.9%). Based on these numbers, we believe we lack the power to detect meaningful changes in downstream outcomes.

© 2022 Ji Y et al. *JAMA Health Forum.*

b) **Total Number of Dialysis Patients.** This is the number of Medicare patients who are on dialysis in either modality. We include all dialysis patients when computing this measure, not just new patients.
c) **Pre-Dialysis Elixhauser Index.** This is the average pre-dialysis Elixhauser index among patients in our baseline sample.
d) **Percentage of Patients Receiving Any Home Dialysis in First 90 Days in 2020.** This is defined the same way as our primary outcome, except that we measure outcomes using data from the year prior to ETC (2020).

3.3 Planned Heterogeneity Analysis

To understand heterogeneity in impact at the patient level, we will conduct the analysis in Aim 1 separately by patient characteristics. Of particular interest is heterogeneity by socio-economic status (which we will proxy for by Medicaid enrollment), and by race. Racial and ethnic minorities and low-income individuals are disproportionately affected by ESRD (Crews et al. 2014, McClellan et al. 2010), they also have significantly lower home dialysis use (e.g., Mehrotra et al. 2016; Shen et al. 2020). In fact, in an effort to reduce these disparities, in July 2021, CMS announced a proposed modification of the program starting in year 2 (2022) that provides additional incentives for improving home dialysis and transplant rates for low-income individuals.[15] About one quarter of our baseline sample is on Medicaid and about one third is non-white.

In addition, we will examine the heterogeneous impact on patients of different baseline propensities to use home dialysis. Specifically, following the method laid out in Einav et al. (2020), we will define $\hat{p}$, the predicted probability of home dialysis use, through a machine learning procedure using data on patient demographics, distance to dialysis facilities, and health status prior to the program. One can interpret this predicted probability of home dialysis use as a compliance propensity score (Follmann 2000). We will document the key demographic, clinical, and other predictors of home dialysis use.

We will then use this predicted probability to divide our baseline sample into subgroups of different propensities for home dialysis, and explore how the ETC program differentially affects those with high vs low predicted probabilities. We will also consider a variant of this predicted probability by incorporating each patient's distance to the nearest dialysis facility into our prediction model.

To understand heterogeneity in impact at the provider level, we will conduct the analysis in Aim 1 separately by characteristics of the dialysis facilities. About 80% of dialysis facilities are for-profit, where two national chains (Fresenius and DaVita) make up the majority of the market share (Walker et al 2010, Shen et al. 2020). Additionally, prior literature has shown suggestive evidence that for-profit facilities tend to be associated with worse patient outcomes (Lee et al 2010). Therefore, we explore heterogeneities by estimating the baseline model separately for (1) for-profit and non-profit facilities, (2) large national chains (Fresenius and DaVita) and others, as well as by (3) facilities' baseline levels of home dialysis rates.

3.4 Planned robustness analysis

[15] https://www.cms.gov/newsroom/press-releases/cms-proposes-changes-reduce-health-care-disparities-among-patients-chronic-kidney-disease-and-end

© 2022 Ji Y et al. *JAMA Health Forum.*

We will examine robustness of our results to alternative specifications. Specifically, we will explore robustness of our results to different controls by estimating versions of equation (1) without controlling for the lagged dependent variable and/or patient demographic and pre-dialysis health. We will also explore robustness of our results to alternative time horizons by estimating equation (1) using 30-day outcomes instead of 90-day outcomes. Finally, since the PPA includes self-dialysis in its calculation of home dialysis rates, we will examine changes in self-dialysis rates and an alternative definition of home dialysis that includes both home- and self-dialysis.

4 Summary Statistics and Power Calculations

We have performed power calculations based on equation (1) using the 100% Traditional Medicare claims data for the dialysis population in 2016. As shown in Table 1, our minimum detectable effect (MDE) size is a 1.98 percentage point difference in home dialysis, our primary outcome. For patient sub-populations the MDE ranges from 2.33 to 3.52.

Table 1: Power Calculation

	Control Mean	MDE	% of Control Mean
	(1)	(2)	(3)
% Any Home Dialysis in First 90 Days	15.58	1.98	12.72
Non-Medicaid	18.57	2.33	12.52
*Medicaid	7.17	2.68	37.35
White	17.12	2.47	14.42
**Non-White	12.54	3.52	28.07
% Dialysis Weeks at Home in First 90 Days	12.98	1.84	14.21
% Dialysis Sessions at Home in First 90 Days	13.26	1.85	13.96

Notes: Power calculation conducted by estimating equation (1) using 2016 Medicare claims data (and lags from 2013). The column "MDE" references the minimum detectable effect size, and "% Control Mean" represents the ratio of MDE over Control Mean. Effect sizes and MDE calculated for an alpha of 0.05 and power of 0.8. Control standard deviations are calculated from residuals of a weighted least squares regression weighting by the average number of new patients in an HRR over the past two years with controls of indicators for Census Region Strata, average patient demographics, pre-dialysis health, and the lagged outcome from 2013. All outcomes are measured as 90-day outcomes, measuring from the first day of dialysis to 89 days afterwards. The sample includes all patients who meet the sample criteria: they are at least 66 years old on the first day of the first month of their dialysis treatment, start dialysis prior to 90 days before the end of 2016, and they are a "new patient" with no dialysis claims for 12 months prior. Please note that the power calculations – unlike our planned analysis - does not include any carrier claims in defining dialysis or previous dialysis use since we only have carrier claims for 20% of Medicare beneficiaries and dialysis claims are almost always recorded in the outpatient file. Sample sizes are N=211 HRRs for control, and N=91 HRRs for treatment, except where noted.

*Sample sizes are N=206 HRRs for control, and N=90 HRRs for treatment.

**Sample sizes are N=197 HRRs for control, and N=86 HRRs for treatment.

© 2022 Ji Y et al. *JAMA Health Forum.*

These strike us as promising MDEs. An increase of 1.98 percentage points or larger for home dialysis seems reasonable to expect from the RCT, given the current low levels of home dialysis use in our study sample (15.6%) relative to its potential. Studies have estimated that about 25-40% of patients would choose home dialysis if presented as an option, and 85% of patients with advanced chronic kidney disease are eligible for home dialysis. However, only one third of patients are currently informed of peritoneal (home) dialysis as an option (Rivara and Mehrotra 2014). It also seems a reasonable effect size given that home dialysis rates are significantly higher in many other countries and regions, such as Hong Kong (74%), Mexico (61%), Guatemala (57%), and New Zealand (47%) (USRDS 2018).

5 Conclusion

This is an analysis plan of the impact of the first year of a nation-wide randomized controlled trial on home dialysis use. Our analysis will help inform the design of health care payment policies for ESRD.

References

Centers for Medicare and Medicaid Services (CMS). 2020. "*Medicare program; specialty care models to improve quality of care and reduce expenditures."* 42 CFR Part 512.

Crews, D. C., Gutiérrez, O. M., Fedewa, S. A., Luthi, J. C., Shoham, D., Judd, S. E., ... & McClellan, W. M. (2014). Low income, community poverty and risk of end stage renal disease. *BMC nephrology*, *15*(1), 1-9.

Einav, Liran, Amy Finkelstein, and Neale Mahoney. 2020. "*Long-term care hospitals: a case study in waste."* Working paper. https://web.stanford.edu/~leinav/wp/LTCH2.pdf

Follmann, Dean A. 2000. "On the effect of treatment among would-be treatment compliers: An analysis of the multiple risk factor intervention trial." Journal of the American Statistical Association, 95(452): 1101–1109.

GAO "End-Stage Renal Disease. Medicare Payment Refinements Could Promote Increased Use of Home Dialysis." October 2015". GAO-16-125.

Lee, D. K., Chertow, G. M., & Zenios, S. A. (2010). Reexploring differences among for-profit and nonprofit dialysis providers. Health services research, 45(3), 633-646.

McClellan, W. M., Newsome, B. B., McClure, L. A., Howard, G., Volkova, N., Audhya, P., & Warnock, D. G. (2010). Poverty and racial disparities in kidney disease: the REGARDS study. *American journal of nephrology*, *32*(1), 38-46.

McCormick, Frank, Philip J. Held, and Glenn M. Chertow. "The terrible toll of the kidney shortage." (2018): 2775-2776.

Mehrotra, R., Soohoo, M., Rivara, M. B., Himmelfarb, J., Cheung, A. K., Arah, O. A., ... & Kalantar-Zadeh, K. (2016). Racial and ethnic disparities in use of and outcomes with home dialysis in the United States. *Journal of the American Society of Nephrology*, *27*(7), 2123-2134.

© 2022 Ji Y et al. *JAMA Health Forum.*

Shen, J. I., Chen, L., Vangala, S., Leng, L., Shah, A., Saxena, A. B., ... & Norris, K. C. (2020). Socioeconomic factors and racial and ethnic differences in the initiation of home dialysis. Kidney medicine, 2(2), 105-115.

United States Renal Data System (USRDS). 2020. *2020 USRDS Annual Data Report: Epidemiology of kidney disease in the United States.* National Institutes of Health, National Institute of Diabetes and Digestive and Kidney Diseases, Bethesda, MD.

Walker, D. R., Inglese, G. W., Sloand, J. A., & Just, P. M. (2010). Dialysis facility and patient characteristics associated with utilization of home dialysis. *Clinical Journal of the American Society of Nephrology*, *5*(9), 1649-1654.

Appendix Table 1: Distribution of New Patients Across HRRs					
	Percentiles				
	5th	25th	50th	75th	95th
New Patients	<11	21	39.5	76	187

© 2022 Ji Y et al. *JAMA Health Forum.*
