# Supplementary material for: Financial Incentives to Facilities and Clinicians Treating Patients With End-stage Kidney Disease and Use of Home Dialysis: A Randomized Clinical Trial
Source: JAMA Health Forum. 2022 Oct 7;3(10):e223503. doi: 10.1001/jamahealthforum.2022.3503 (PMC9547325; doi:10.1001/jamahealthforum.2022.3503)
Supplement: Supplement 3. — Data sharing statement [file jamahealthforum-e223503-s003.pdf]

Data Sharing Statement

Ji. Financial Incentives to Facilities and Clinicians Treating Patients With End-Stage Kidney Disease and Use of Home Dialysis. *JAMA Health Forum*. Published October 07, 2022. doi:10.1001/jamahealthforum.2022.3503

Data
Data available: No

Additional Information
Explanation for why data not available: Our paper uses confidential administrative data, which cannot be made available online (although there exist established processes for applying for data access). The data are provided by the Center for Medicare and Medicaid Services (CMS). There is a standard application process (described in more detail here: http://www.resdac.org/). In our experience, we have not had trouble getting approval as long as we provided an adequate data security plan and explained why the files we were requesting were needed for the research we were proposing (and of course paid for the data).
